# Piloting a low-cost hardware intervention to reduce improper disposal of solid waste in communal toilets in low-income settlements in Dhaka, Bangladesh

**DOI:** 10.1186/s12889-017-4693-x

**Published:** 2017-08-29

**Authors:** Farzana Yeasmin, Stephen P. Luby, Ronald E. Saxton, Fosiul A. Nizame, Mahbub-Ul Alam, Notan Chandra Dutta, Abdullah-Al Masud, Dalia Yeasmin, Anita Layden, Habibur Rahman, Rachel Abbott, Leanne Unicomb, Peter J. Winch

**Affiliations:** 1 Environmental Interventions Unit, Infectious Disease Division, icddr,b (formerly, International Centre for Diarrhoeal Disease Research, Bangladesh), Dhaka, 1212 Bangladesh; 2 Infectious Diseases and Geographic Medicine, Woods Institute for the Environment, Stanford University, Stanford, CA 94305 USA; 3 Department of International Health, Johns Hopkins Bloomberg School of Public Health, Baltimore, MD 21205-2103 USA; 4 Water and Sanitation for the Urban Poor, London, EC4V 6AL UK; 5 Water and Sanitation for the Urban Poor, Dhaka, 1205 Bangladesh

**Keywords:** Urban sanitation, Fecal sludge management, Communal toilets, Waste disposal, Low-income community, Toilet functionality

## Abstract

**Background:**

Bangladesh faces daunting challenges in addressing the sanitation needs of its urban poor. Maintaining the cleanliness and functionality of communal toilets is dependent upon periodic emptying of fecal sludge, and cooperation between users of communal toilets. Trash disposal into latrines can block the outflow pipes, rendering the toilets non-functional.

**Methods:**

Pre-intervention: We conducted in-depth interviews with five operators of fecal sludge emptying equipment and five adult residents who were also caregivers of children. We identified factors contributing to improper disposal of trash into communal toilets, a barrier to operation of the equipment, in low-income communities of Dhaka, Bangladesh.

Intervention design: We developed behavior change communication materials to discourage waste disposal in toilets, and promote use of waste bins. We conducted six focus group discussions with adult male, female, landlord and children to select the preferred design for waste bins to be placed inside toilets, and finalize communication materials.

Post-intervention: We then pilot-tested an intervention package to promote appropriate trash disposal practices and thus facilitate periodic removal of fecal sludge when the latrine pits become full. We conducted 20 in-depth interviews and four focus group discussions with community residents, landlords and cleaners of communal toilets.

**Results:**

Barriers to appropriate waste disposal included lack of private location for disposal of menstrual hygiene products, limited options for formal trash collection and disposal, and the use of plastic bags for disposing children’s feces. A pilot intervention including behavior change communication and trash bins was implemented in two urban slum communities. Spot checks confirmed that the bins were in place and used. Respondents described positive improvements in the appearance of the toilet and surrounding environment.

**Conclusion:**

The current practice on the part of local residents of disposing of waste into toilets impedes the safe removal of fecal sludge and impairs toilet functionality. Residents reported positive changes in toilet cleanliness and usability resulting from this intervention, and this both improves the user experience with toilets, and also promotes the sustainability of the entrepreneurial model of Vacutug operators supported by WSUP.

## Background

Bangladesh is rapidly urbanizing, a process driven not only by economic opportunities in urban areas, but also displacement of the rural poor due to environmental degradation and climate change [1]. In this study we examine one aspect of Bangladesh’s urban health challenge: how to maintain the cleanliness and functionality of communal toilets, and barriers to periodic emptying of fecal sludge with portable vacuum machines. This effort represents only one piece of a larger set of solutions that must be put in place to improve urban sanitation. Nevertheless, we will argue that the iterative methodology for identifying problems and assessing potential solutions under conditions of crowding and scarcity is applicable to a number of other sanitation challenges in low-income urban communities.

Nearly 15 million of Bangladesh’s inhabitants live in the capital city, Dhaka, of whom approximately six million reside in urban slums and 4.3 million use communal toilets (toilets shared by multiple households) as their primary means of sanitation [2]. There is evidence of a potential increased risk of undesirable health outcomes in shared toilet facilities compared with individual household toilets [3]. Residents of low-income communities struggle to maintain communal toilet cleanliness and functionality due to a wide variety of factors including a lack of social cohesion, technological problems, and poor governance [2]. A study conducted in Orissa, India found that shared facilities were less likely to be functional, less clean, and more likely to have feces compared with individual household toilet [3]. A study conducted in Indonesia and Bangladesh found that users of shared facilities often reported feeling both satisfied and safe when sanitation facilities were clean and shared by a limited number of households [4]. Research in Orissa, India documented that over half of the users of the communal latrine facilities were unaware when the tank was last emptied [5]. A major barrier to the functionality of latrines in urban slums in Dhaka is lack of fecal sludge management [6]. Currently when latrines fill up, they are either abandoned, or emptied manually [6–8]. Manual emptying is common in many settings: destroying the squatting slab and digging the sludge out with hand tools such as spades, shovels and buckets [7, 8]. However this practice/method contaminates the surrounding environment, and exposes the emptiers to high concentrations of pathogens. Another way of emptying is vacuum-based method which uses powered air flow to suck pit contents through a hose into a container under a partial vacuum [6].

In Dhaka, installation of sanitary sewer systems is not a viable solution in the short term. The non-governmental organization Water and Sanitation for the Urban Poor (WSUP) is recruiting, training and equipping operators to perform emptying of fecal sludge in low-income communities with one type of vacuum-based equipment, Vacutugs [9, 10]. The vision is that these Vacutug operators will charge for their services, and that this fee-for-service model will represent a financially sustainable solution for fecal sludge management.

The entire Vacutug entrepreneur model is put at risk when slum residents dispose of trash in latrines. It blocks the Vacutug machines, making them inoperable [9, 10]. The Vacutug operator incurs costs to repair the machine, putting at risk the financial model of working as a Vacutug operator. An assessment of fecal sludge removal in WSUP’s intervention area found that only 10% of fecal sludge in low-income communities in Dhaka was removed by operators using Vacutug or an alternative device for emptying fecal sludge such as a diaphragm or Gulper (Rahman et al., Unpublished data). Fifty-seven percent was removed by manual operators and 33% of latrines remained un-emptied [11].

We undertook formative research to identify factors affecting disposal of obstructive trash in communal toilets, and to inform the development of feasible and appropriate interventions to promote appropriate trash disposal practices among users of communal toilets in a low-income community in Dhaka, Bangladesh. Our formative research followed three phases leading up to a multi-component, behavior change intervention to improve usage and functionality of communal toilets. We structured our qualitative interview guides according to the three dimensions of the IBM-WASH framework to address influential behavioral factors along contextual, psychosocial and technological dimensions [12]. IBM-WASH is a Social Ecological Model, with rows representing the nested levels where actions need to be taken, and the three columns or dimensions serving to identify different categories of factors that need to be considered when developing WASH interventions.

The objectives of this paper are to:

1. Explain the reasons for deposition of solid waste in toilets, contributing to toilet blockage,
2. Describe the development of an intervention to prevent blockage of toilets, and
3. Discuss the lessons learned from pretesting of the intervention and make programmatic recommendations to apply these insights more broadly.

## Methods

To better understand waste disposal practices and fecal sludge management in low-income, urban communities in Dhaka, we selected two communities, Bauniabad and Kolyanpur, where Vacutug pumps supplied by WSUP had become blocked because of obstructive solid waste disposed directly into toilets. IBM-WASH had guided analysis of qualitative data in a number of studies in Bangladesh. IBM-WASH guided the design of codes, thematic analysis, and organization of emerging themes by levels and dimensions in the framework. The authors analyzed their results according to the contextual, psychosocial, and technological dimensions, at the habitual, individual, household, and community levels, related to behavior in infrastructure-restricted settings [13]. In another study, the authors developed the Cholera-Hospital-Based-Intervention-for-7-Days, and the design of the intervention was informed by factors from the IBM-WASH model and constructs from the Health Belief Model [14]. In another study, the authors mentioned that the study team used both a priori codes based on the IBM-WASH model and emergent codes [15].

### Phase 1 (pre-intervention): Identifying behaviors to improve waste disposal practices

The field team conducted in-depth interviews with five operators of Vacutugs and similar vacuum equipment who performed fecal sludge emptying to understand the barriers to fecal sludge management. Three operators were hired by WSUP and one each was selected from among operators hired by two other NGOs: Dustho Shastho Kendro (DSK) and Population Services and Training Center. All had performed recent sludge removal. Operators identified toilets in which trash had been improperly disposed into the pit. In close proximity to these toilets, we purposively sampled two adult male and three adult female residents who were also caregivers of children under two, because bags of children’s feces had been detected as causing blockages. We conducted in-depth interviews with these residents regarding trash creation and disposal practices and barriers to appropriate waste disposal and communal toilet maintenance. The in-depth interviews lasted 30–60 min. These data were used to design materials for a pilot intervention addressing barriers to appropriate toilet waste disposal.

### Phase 2 (intervention design): Developing and pretesting behavior change materials

We developed behavior change communication materials to discourage waste disposal in toilets, and examined four potential waste bin hardware models to facilitate adoption of appropriate waste disposal behaviors. In Phase 1, we characterized the problem. In Phase 2 we selected hardware (waste bins with lids), behavioral recommendations (disposal of items into bins that could otherwise block toilet outflow), and visual aids to communicate the behavioral recommendations: stickers (for waste bins and latrine doors), signs (for walls of compounds) and cue cards (for interpersonal communication by promoters). The choice of visual aids to be developed was influenced by our desire to place the information as close as possible to where the behavior would be practiced, i.e. near the toilet. We assessed the acceptability and preferences for four candidate waste bin models to facilitate adoption of appropriate waste disposal behaviors. We then conducted six focus group discussions—three with female residents and landlords, two with male residents and landlords, and one with children—to select the preferred waste bin design and elicit feedback on the stickers, posters and cue cards before selecting and finalizing intervention hardware and print materials for the pilot intervention. We selected the participants who were available at the time of focus group discussion, after informing landlords or compound managers prior to the focus group discussion and asking them to remind their tenants. The focus group discussions lasted 60–130 min. In these communities there are two kinds of landlords. One kind lives elsewhere in the city. They were not part of the focus group discussions. Another kind of landlord lives in the same compounds. They use the same toilets. In this slum context, this second type of landlord has similar socio-economic status to the tenants. It is common in this context that tenants are at liberty to share their own opinions in the presence of landlords and often they discuss with landlords their problems with use and maintenance of the latrines. We encouraged the landlords to be present in the focus group, because their participation would facilitate eventual decisions over assignment of roles and responsibilities among compound members, such as emptying of waste bins. Landlords can play a pivotal role for maintenance so it was important for us to ensure their presence in focus group discussions.

### Phase 3 (post-intervention): Conducting and assessing waste disposal pilot intervention

**Fig. 1:**
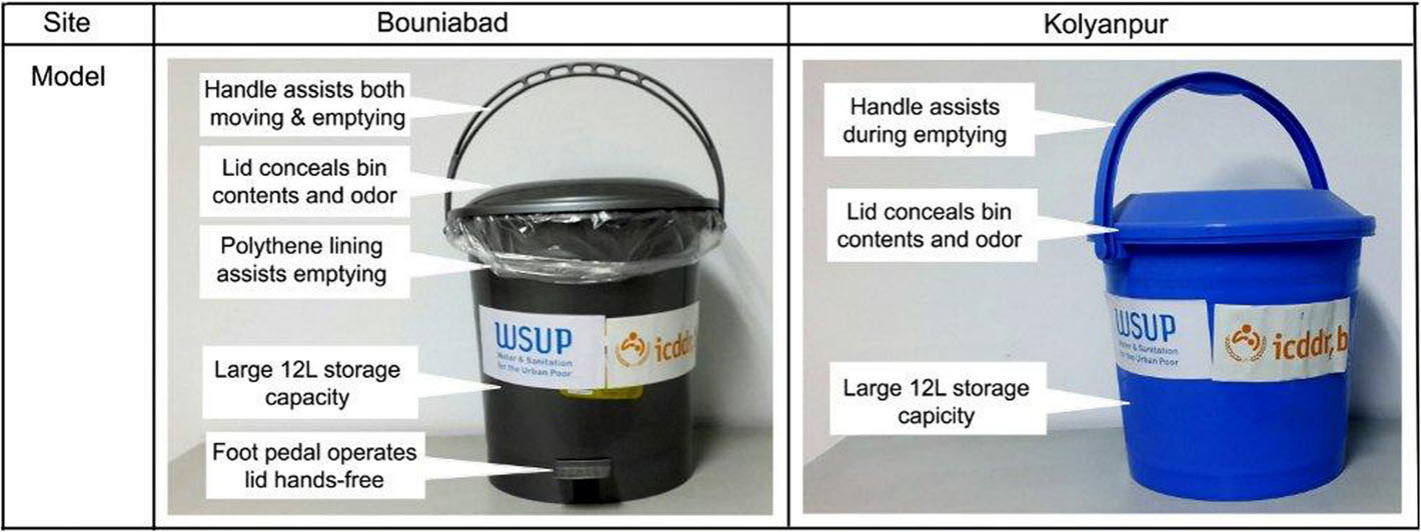
Description of hardware used for pilot toilet waste disposal intervention

**Fig. 2:**
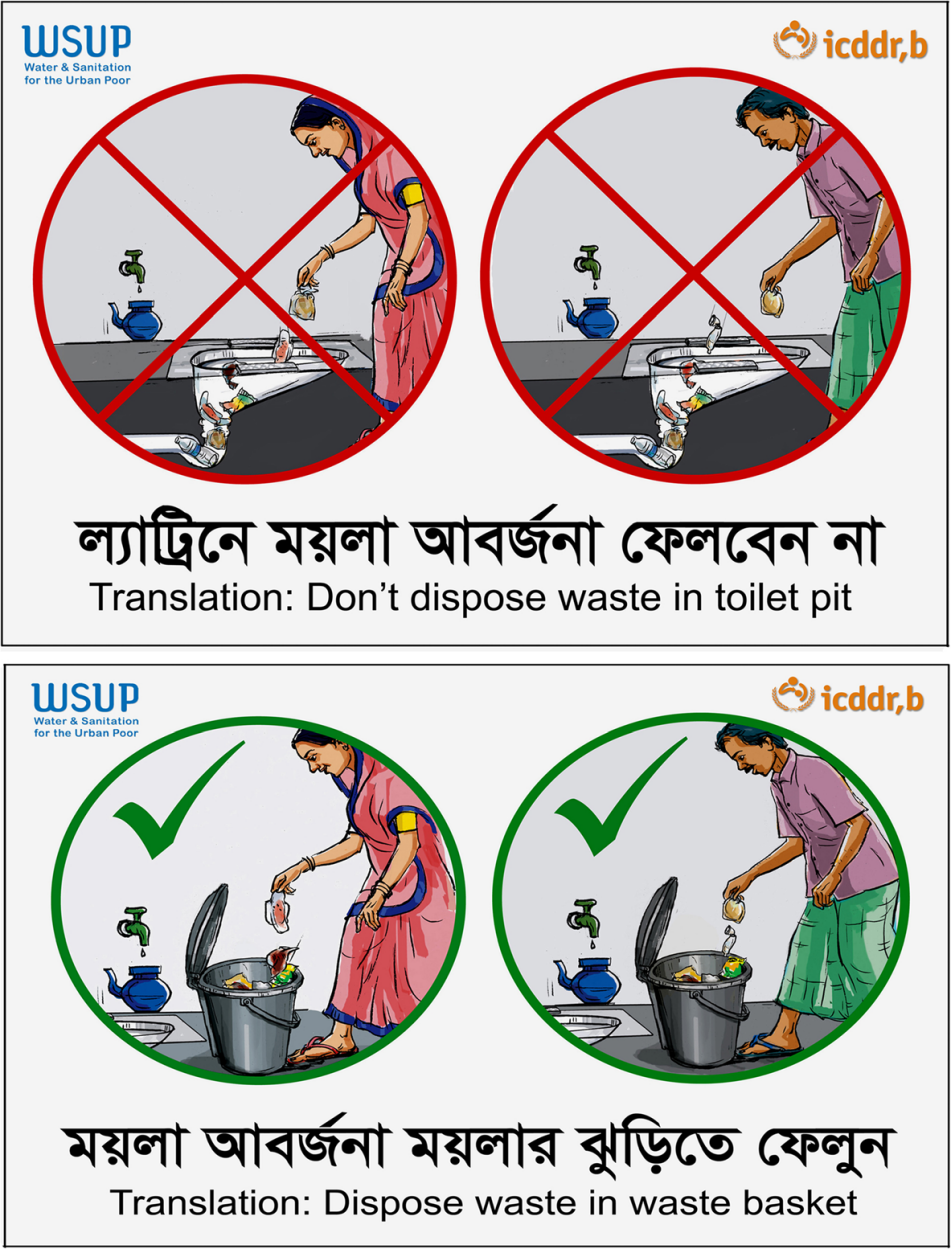
Sample of behavior change communication materials

We promoted the pilot intervention including hardware and behavior change messages at two communal toilet sites in Bauniabad and Kolyanpur. The pilot intervention tested one particular one waste bin model in one site and another particular model at the other site (Fig. 1). Signs indicating appropriate and inappropriate waste disposal behaviors were posted inside communal toilets as a cue to action (Fig. 2). We conducted courtyard sessions to introduce hardware, present behavior change messages, demonstrate how to use the hardware to encourage target behaviors, and to recommend that the communities organize a system for emptying and maintaining bins. Weekly, household interpersonal communication sessions reinforcing the behavior change messages and daily spot checks of the bins were conducted for a 2-week period. To explore the acceptability and feasibility of the pilot intervention, we conducted two follow-up qualitative assessments which included 24 in-depth interviews at four weeks and ten weeks after the pilot commenced. Each assessment included 12 in-depth interviews- five with male communal toilet users who were available and willing to participate, five with female communal toilet users who were of reproductive age and agreed to participate, and two with waste bin emptiers. One site (Bauniabad) had a toilet comprised of three cubicles and the other site (Kalyanpur) had two toilet cubicles. We selected one adult male and one adult female toilet user per toilet cubical according to their availability and willingness to participate for each assessment. We identified and selected waste bin emptiers based on who at the time of interview was currently responsible for keeping the waste bin clean and their availability to participate. Assessments also included an additional focus group discussion with the female toilet users at each site (see Table 1 for a summary of data collection). We reached thematic saturation after completion of these 24 interviews.

**Table 1:** Summary of data collection procedures

Phase	Method (sample)	Main objective	Type of data collected
*Phase 1*: Identifying barriers to fecal sludge management and target behaviors to improve waste disposal practices	(10) In-depth interviews (5 Vacutug operators; 3 female residents with child <2 years; 2 male residents with child <2 years)	To explore knowledge, perceptions, reported practices and barriers to fecal sludge management and waste disposal	Demographic characteristics of respondents, problem of usage toilet, existing practice of toilet use, way to keep toilet clean
*Phase 2*: Selecting and pre-testing behavior change communications and materials for waste disposal pilot intervention	(6) Focus group discussions (13 male residents/landlords; 12 female residents/landlords; 14 children)	To refine and select final behavior change communications and hardware for pilot intervention	How to improve the messages and the materials: content, characteristics and understandability of messages and size, color, shape, benefits of hardware
*Phase 3*: Conducting and assessing waste disposal pilot intervention	*Pilot*: Hardware provision, BCCs, courtyard sessions, household interpersonal communications, spot checks of bins *Assessments*: (24) In-depth interviews (4 bin emptiers; 10 female residents/landlords; 10 male residents/landlords) & (2) Focus group discussions (18 female residents/landlords)	To explore knowledge, perceptions, reported practices and barriers to keep toilet clean, and to identify problems in using waste bin and practices for emptying and maintaining the bin	Knowledge and practice of waste disposal, attitude toward disposing waste in bin, barrier of using bin, motivational affect in promoting disposal of waste in bin

### Data analysis

All data were collected by native Bengali speakers with extensive qualitative research experience. Audio recorded data from in-depth interviews and focus group discussions were transcribed verbatim in Bengali and then translated to English text in Microsoft Word. transcriptsin The final English transcripts contained numerous transliterations so as to retain the original tone of the interviews. The translators were instructed to transliterate great portions of the interviews which contained local terms and expressions. Based on themes we created codes which were chosen prior to data collection according to the study objectives. The research team met regularly during data transcription and translation to aid data familiarization. We generated additional inductive codes from the data. Individual and group interview transcripts were then manually coded and categorized according to these major codes.

Although the interviews were coded and categorized individually, the research team drew inferences from the findings collectively. The research team also took additional open ended field notes even when they were not officially observing or interviewing. These included informal discussions and observations. The team noted the tone and attitudes of the respondents during data collection and met regularly during data transcription to generate additional relevant codes and themes.

We developed data collection and coding guides based on our research objectives and using the IBM-WASH model [12] to emphasize influential behavioral factors associated with waste disposal practices along the contextual, psychosocial and technological dimensions. After coding, the field team translated the coded data into English. We analyzed each in-depth interview and each focus group discussion separately and in the findings we have drawn inferences collectively.

### Ethical considerations

We obtained written informed consent from the adult study participants as well as verbal permission to conduct intervention activities from the landlords in the pilot communities. We secured assent from children and obtained consent from parents to conduct the focus group discussions with children. The study protocol was reviewed and approved by the ethical review committee of icddr,b and institutional review board of Stanford University.

## Results

### Barriers to fecal sludge removal with Vacutugs

The operators who perform fecal sludge emptying with Vacutugs reported that fecal sludge contained rags, glass bottles, plastic bottles, plastic bags, packets of detergent and shampoo, children’s feces wrapped in plastic or placed in plastic bags, children’s wash cloths, pads and pieces of cloth used for menstrual management, condoms, toys, soap cases, broken brushes and construction debris. If they do not manually remove these items before emptying liquid sludge, their machine collection pipes clog. They stated that the community is largely unaware that these items cause blockages when disposed into toilets, nor that there are increased time and labor costs incurred for removal. Vacutug operators suggested communicating the consequences of inappropriate waste disposal to residents and restricting toilet access of those who fail to change behavior.

### Waste disposal practices of residents

Male and female residents described how they dispose of their household solid waste. Although interviewed separately, male and female residents reported similar categories of waste-related topics including 1) management and disposal of children’s feces, 2) disposal of items used for personal hygiene, 3) disposal of items used for menstrual management and 4) debates concerning the costs of waste management.

Households dispose of daily waste, such as food waste, into dumpsters, which then are emptied by the city’s Solid Waste Management system. As caregivers for children under two, respondents also managed disposal of children’s feces, because young children often defecate within or around the household. The caregivers did not want to take the child to the toilet because children were afraid to enter the dark latrines and because it was inconvenient to bring the child to the latrine. They reported that children’s feces are placed into plastic and paper bags or collected in potties, and subsequently disposed of nearby in lakes, bushes, or toilet pits.

Residents reported that people use rags, cloth, mud or pieces of bricks in place of more expensive tissue paper to dry themselves after cleaning themselves with water following urination and defecation. These various items used for personal hygiene are sometimes disposed of into toilets after use.

Female respondents mentioned that they usually use cloths for menstrual management, as disposable pads are expensive. They stated that older women are more likely to wash and reuse the cloth compared to younger women, who are disgusted after using a cloth and dispose of them after one use. They dispose of used cloth in drains and ditches. However, some women are reticent to dispose of these items in public places, and therefore dispose of them in the toilet. Male residents similarly reported that women dispose of menstrual pads and cloth in toilets and sometimes wrap them in plastic in order to hide them, due to embarrassment.

Residents identified that municipal solid waste management services were available to serve low-income communities, but at a cost. For this reason, some residents do not want solid waste management to serve their communities, while others believe that these services are necessary to maintain a healthy and clean environment and prevent indiscriminate disposal of waste in open spaces where children can be exposed. It is a usual practice in Bauniabad to dispose of waste in a nearby lake, as a 27-year-old male respondent said, *“Ultimately we dispose of the trash into the lake, as we have a lake beside our residence. Other people also dispose of their trash in that area. Easily we dispose of our trash into the lake.”* The residents of this neighborhood questioned why they should pay the waste collector who disposes of the waste into the same lake, when they could accomplish this action on their own.

### Responses of residents to the intervention

To discourage disposal of various solid waste items in the toilet pit, we installed waste bins inside the toilet cubicles. We posted signs indicating correct use of the bins and emphasized that disposal in the toilet could cause blockages. The bins were well accepted and used at both pilot sites (Table 2). During spot checks, field research assistants found disposed rags and sanitary pads in the waste bins. The waste bins were emptied regularly. Focus group respondents said that the intervention reduced toilet blockages. All household members found the bins easy to use and even children were interested to use them. The location of bin and signs inside the cubicle provided good reminders of appropriate disposal, and motivated use. Respondents said that the bins had adequate volume to meet community trash needs. A 41-year-old male respondent from Bauniabad spoke of the convenience of the bin, “*When we go to the toilet in that time, it works as a reminder to dispose waste in that bin. No need to go to the lake side to dispose waste in that area.”*

**Table 2:** Toilet user and bin cleaner perceptions of pilot intervention

Benefits at both sites	Barriers at both sites
+ Reduced toilet blockages + Bin has adequate volume for community trash needs + Signs with pictures and text clearly illustrate expected use + Location of bin and signs inside cubicle provides good reminder of appropriate use + Lid provides privacy and masks odors	- Certain items (sanitary napkins, condoms) require wrapping before disposal to prevent user shame and caretaker disgust - Need to engage community leaders (landlords, teachers, Imams) to settle maintenance disputes and instill importance of bin use
Perceptions specific to Bauniabad (Ash-colored plastic bin with foot pedal to open it)	Perceptions specific to Kolyanpur (Blue-colored bin with lid without foot pedal)
+ Foot pedal prevents disgust of having to touch lid with hands + Path leading to toilet free of trash - Some residents refuse responsibility to empty bin - Waste bin maintenance system was difficult to enforce in community without a paid toilet caretaker	+ Bin color is attractive and noticeable reminder to use + Caretaker found cubicle to be less disgusting to clean - Parents must instruct children not to damage bins or signs - Disgust to open lid with hands because of urine splatter on lid

Signs posted inside the toilet cubicle with both pictures and text clearly illustrated expected use. A 45-year-old female respondent from Bauniabad said,*"I like the signs because from this sign, people have come to know that if they dispose of the waste [in the] toilet [it] may block or jam. Messages were written in this sign and it is possible to read them while using the toilet."* Use of the bins improved the toilet environment, *"When the bucket was not in the toilet, people used to throw waste [into] the toilet pit. There was bad odor all around the toilet that made me feel bad."* Most female respondents said that they liked that people can throw waste in the bin so that the toilet does not get dirty. A few also said they felt comfortable using the bins to dispose of cloths used during menstruation. Some respondents reported that the waste bin reduced their chance of getting diseases.

### Disgust as a barrier to using trash bins inside toilets

Both male and female respondents in the first assessment said that certain items like menstrual rags or condoms require wrapping before disposal to prevent feelings of disgust. Male discomfort with menstruation and opposition to disposal of menstrual products in waste bins was also a major barrier for women to use the bin. A 31-year-old Bauniabad man said, *"I don’t know whether women are embarrassed to dispose rags and napkins in that bin, but I feel disgust whenever I see any napkin or rags visible anywhere whether it is inside the bin or in other places. It’s a matter of embarrassment. Although this bin has a lid, whenever we open it, I feel disgust. This should be a confidential matter as it is an embarrassing matter."* Similarly, a 25-year-old female respondent said that, *"Women use small cloth pieces during their menstrual period and throw those in the waste bin which was set in the toilet. But I personally never dispose waste into the bin. I use the cloth again and again washing it with water. I do not dispose in the bin because I feel disgust (ghrina) as everyone throws menstrual pads in the bin."*

Some female focus group participants also expressed the perception that they could spread disease if men saw their openly disposed menstruation rags. *"We believe many diseases are spread from rags and napkins to male persons if they see them. It affects eyesight. We call that 'Black visual disease' (Kali drishti beram). That’s why we do not like to dispose used rags openly."* A few female focus group respondents reported that they wrap used rags with polythene or paper before disposing them in the bin, because they feel embarrassed. In Kolyanpur, where we provided the traditional bucket waste bin model, a small number of respondents reported feeling disgust to open the lid with their hands because of urine splatter on the lid.

### Bin maintenance problems

In Kolyanpur, a hired cleaner took the responsibility of empting and cleaning the waste bins, and collected 39 US cents per month from each household for this service. In Bauniabad, there was no paid cleaner and three women took on these responsibilities voluntarily. In the absence of a paid cleaner, bin maintenance was more difficult. A 31-year-old male respondent of Bauniabad said, *"In our compound, maintenance of this bin is a problematic issue. No one wants to empty these waste bins. Some stubborn people don’t want to use these and clean these. After every 5 to 6 days, waste bins are being cleaned. But most of the time no one wants to take the responsibility to clean and maintain these waste bins. If all users share these responsibilities and do it voluntarily, then maintenance would be easier."* When bins were emptied fewer than three times per week, residents reported that they created a foul odor.

The female cleaner from Bauniabad said that the intervention should engage respected community leaders, suggesting landlords, teachers, or Imams could help to settle maintenance disputes and instill the importance of bin use. She said, *"It [the intervention] could be better if you will talk with [the] landlord. Then [the] landlord will talk [with] other tenants. This could reduce quarrels (among renters) or they can help if a conflict arises."*

## Discussion

Sridhar recently has argued that urban poverty is much more difficult to address than rural poverty, and yet research on how to address urban poverty received comparatively much less attention [16]. We draw on the Integrated Behavioral Model for Water, Sanitation and Hygiene (IBM-WASH) as a framework for identifying lessons learned from development of our intervention for promoting cleanliness of communal toilets that might be applicable to other health problems occurring among urban populations living in poverty [12].

The first dimension in IBM-WASH is the contextual factors. These need to be taken into account in developing interventions, but are not readily amenable to change. In low-income communities in Dhaka, contextual factors at the community level include insecurity of land tenure, control of land and resources by local landlords and power-brokers (*mastaan*), and the limited political power of the urban poor [17]. More specific to this intervention is the weak and limited disposal system for solid waste, the lack of a sewer system and lack of wastewater treatment facilities. People from these communities dispose household trash directly into surrounding lakes and lowland areas due to lack of services and poverty. The common features of a slum include poor-quality housing, limited educational and social services, and lacking or limited water, sanitation, electrical grid and street network [18]. Again most of the slums are located near polluted water bodies, swamps, ditches or putrid drains [18]. A study conducted in Kampala, Uganda has revealed that group discussions are effective in improving the cleaning behavior of shared sanitation users [19]. In our study, we conducted baseline and final household surveys where we found the intervention households had less likely to have the shared toilet with visible fecesinside the pan than the control households [20]. Various non-state actors such as landowners and non-governmental organization (NGOs) fill some but far from all of the gaps in regulation and service provision [17]. There is little hope of better governance in the near term [17]. In our study we had also demonstrated low-cost water storage and flushing hardware and promotion of toilet maintenance behaviors which we found as reinforcement of improving both hygienic conditions and user satisfaction with shared toilets even in the area which had water scarcity. And we found both the residents and the landlords were pleased with these efforts [21]. Linking the interest of entrepreneurial Vacutug operators who want to keep their machines from clogging with users of shared toilets who want clean facilities provides an opportunity for sustainable improvement. This project did not attempt to address the ineffective waste disposal system in Dhaka’s urban slums. Funding trash management removal and treatment is necessary for all countries to attain a sanitary living environment. In addition to funding resources, it is important for political leadership to be interested in improving living conditions in these settings. However, political leadership may benefit financially from keeping services at poor levels, as is the case with local leaders (*mastaan*) who control power over basic utilities and lands and, in return for controlling these resources, profit from demanding a daily fee from the local businessmen [17]. Public authorities in charge of water supply through local government bodies exert their political power all the way down to the local level to control these basic resources [22, 23].

Psychosocial factors are the second dimension in the IBM-WASH framework. Psychosocial factors identified in this study were gender roles, disgust and shame related to waste products, specifically wastes related to menstrual management and understanding the consequences of trash disposal in the toilet. Technology factors are the third dimension in the IBM-WASH framework. In this case, the technology included the design and construction of existing toilets, and the waste bins introduced by the project (Table 3). The project team was able to assemble an intervention package that addressed psychosocial and technology factors, despite the challenging contextual factors that placed constrains on implementation.

**Table 3:** Implications for design of interventions to promote cleanliness of communal toilets, organized around the three dimensions in the IBM-WASH model

Dimension in the IBM-WASH model	Implications for intervention design
Contextual dimension in the IBM-WASH model	
Contextual-level barriers • Access: Lack of waste disposal options in slums influences improper disposal of waste in toilet pit • Geography and Income: Solid waste management in slums is complicated by geography and cost, and residents may be less willing to pay for what they perceive to be poor service • Unfavorable environment for habit formation: Systems for cooperative toilet maintenance are complicated by resident transience in urban slums	• Low-cost hardware and behavior change interventions can promote appropriate waste disposal practices and facilitate safe fecal sludge removal • Feasible and environmentally sound waste collection systems should be explored • Landlords can be engaged as a more permanent element to enforce toilet maintenance systems; paid cleaners may more reliably maintain hygienic conditions of communal toilets, including emptying of solid waste than resident volunteers
Psychosocial dimension in the IBM-WASH model	
Psychosocial-level barriers • Privacy: Lack of private space for menstrual management encourages disposal of menstrual hygiene items in toilet pit • Shame & Disgust: Community feels disgust and embarrassment when encountering items used for menstrual management disposed openly • Existing habits: Caregivers are accustomed to collecting and disposing of children’s feces using plastic bags, but disposal of these bags in the toilet pit impairs fecal sludge removal	• Promote disposal of items used for menstrual management in a closed bin • Promote wrapping of used menstrual hygiene items with locally available materials prior to disposal • Child potties may be promoted to ensure safe disposal of children’s feces without impairing fecal sludge emptying
Psychosocial-level facilitators • Shared Values: Residents had a strong shared value for toilet cleanliness and worked to maintain the good condition and location of waste bins	• In areas where hardware theft is a concern, messages should emphasize toilet cleanliness as a shared value to build collective efficacy to maintain the good condition and location of waste bins
Technology dimension in the IBM-WASH model	
Technological-level barriers Convenience: Community members disposed of items used for personal hygiene in the toilet pit at the point of use • Strengths and weaknesses of the hardware: Although both bin models were well-accepted, some residents felt reluctant to dispose of waste when they had to touch the bins in order to use them	• Waste bins were conveniently located to facilitate habitual use; • Ease of operation, durability, adequate volume, and attractive color of bins are attributes to consider in selecting waste-disposal facilitating hardware that is well perceived

### Technology: Provision of waste bins

Adult men and women disposed of items used for personal hygiene in the latrine pit at the point of use, which suggests that convenience was a factor contributing to the inappropriate disposal of trash. Our intervention addressed this issue by placing bins inside the toilets and ensuring bin capacity was adequate to accommodate daily trash. Residents remarked that the attractive color of the bin acted as a reminder for use. Color, size, and location should be considered in selecting bins for trash disposal interventions, such that hardware is perceived to be attractive and convenient to use, thereby facilitating habit formation. During feedback on bin design from community residents, finding a convenient way of disposing of child feces remained an important concern.

We promoted use of trash bins – by advertising their use through signage and demonstrations and placing them in convenient locations in the toilets. Future studies may examine the issue of price, and explore a self-sustaining way of paying for the bins and their maintenance. Additionally, future interventions ought to consider the provision of child potties to ensure the convenient disposal of child feces.

### Psychosocial factors: Community and household levels

Lack of social cohesion and poor sanitation system governance in urban slums have been identified as the principal barriers to communal toilet maintenance in Dhaka [2]. One of the strategies that the current intervention employed to increase social cohesion was to place behavior change messages in common visible locations to encourage discussion. Moreover, the strategy involved community stakeholders in the decision making process for bin emptying-- to appoint a paid cleaner or volunteers. This potentially enhanced social cohesion by encouraging dialogue between community participants. A previous study of communal toilet users in Kampala, Uganda found that if people spend time talking to each other about toilets, they were more likely to work together to keep them clean [24]. In Kampala people who considered keeping the toilet clean to be important were more likely to be involved [24]. In our study, we illustrated the hazards of disposing of waste in the toilet (i.e. clogs, increased expense to empty) using behavior change materials in order to increase perception of the importance of using the waste bins. Users expressed that they found these materials to be useful and informative. Users had a strong shared value for the hardware and messages. Ensuring a stable context, by guaranteeing that the waste bins are present and functional, is critical to elicit habitual behavior [25].

This study tested two models of bin maintenance to assess cleanliness and sustainability. In one model the bins were emptied by female volunteers and in the other model someone was paid by the compound members to empty the bins. The toilets with paid bin emptiers remained much cleaner than the toilets maintained by volunteers, which makes toilets with paid bin emptiers a much more potentially sustainable model. In the Indian cities of Pune, Mumbai, Kanpur and Bangalore, community managed toilet blocks demonstrate self-sustainability of community-run pay-to-use toilets constructed by urban organizations. They found it possible to employ someone for full-time to clean toilets, keep water tanks filled and collect a charge from outsiders [26].

### Psychosocial factors: Individual and habitual levels

We addressed individual level psychosocial factors such as limited knowledge, skills and self-efficacy [27] by posting signs depicting the correct use of the waste bins and by demonstrating and role modeling the recommended behaviors. According to the concept of observational learning, a construct described in Social Cognitive Theory, individuals learn new behaviors by watching others perform them [28]. The signs related to waste disposal were developed in response to their recommendation to provide instructions on how to dispose waste properly and perceived by residents to be readily understandable.

A need for privacy was a major factor that influenced the disposal of menstrual management waste in toilets. Participants were embarrassed to dispose menstruation products which could be seen, and even spoke of fears that the sight of menstruation products could cause sickness. Such concerns are particularly relevant in Bangladesh, where menstruation is traditionally considered a time when women are “polluted” and therefore should maintain distance from others, in addition to it being an extremely private matter [29]. Despite the bins having lids, residents and toilet caretakers designated to empty the bins felt uncomfortable when they encountered sensitive items like menstrual rags disposed in the bin. We found that some women wrapped used rags with polythene or paper before disposing in the bin. This practice may be necessary to create an additional layer of privacy for the disposer as well as to prevent disgust for subsequent bin users or the caretakers who empty the bin contents.

Menstrual management was an important component of waste management for this community but is largely neglected by WASH programs in South Asia [30]. There is a need to develop long-term strategies for improving women’s sanitation options. To address the privacy/shame issues detected in this study, a bin with a flap rather than a lid that cannot be opened except by the disposer to conceal bin contents could be considered.

Each of the pilots took place over the course of one month, which limited our ability to assess the durability of the hardware and behavior over a long period of time. We did not conduct separate meetings with landlords regarding the enforcement of maintenance systems. This addition would be important for developing a robust mechanism for emptying waste bins in case volunteers stopped maintaining them. Additionally, we conducted pilots in only two compounds, which may not be representative of all urban slum sites in Dhaka. A larger trial involving 600 compound is currently being implemented by iccdr,b and WSUP, which will demonstrate whether an intervention package that includes waste bin provision is effective in improving toilet cleanliness and usability.

## Conclusions

This intervention to improve waste disposal in communal toilets in this settings demonstrated that a lidded waste bin inside the toilet with removable plastic bag, behavioral recommendations for what items are to be placed in the waste bin, visual aids and interpersonal communication to promote the behavioral recommendations and assignment of responsibility for regular emptying of the waste bins reduced the improper disposal of waste in the toilet pit that can impede the safe removal of fecal sludge and impair toilet functionality. Residents reported positive changes in toilet cleanliness and usability resulting from this intervention. Residents liked the waste bins because they were used and the toilet cubicle remained clean and orderly. We observed a strong shared interest in maintaining the waste bins, encouraging their continued maintenance.

Designation of a site or a collection service for disposal of materials placed in the waste bins remains challenging and we still have not identified a satisfactory solution to it. People dump waste in lakes, ponds, ditches etc. in areas where there is no system in place for regular solid waste collection.

Despite the success of this short intervention, we observed a number of ways in which waste disposal in communal toilets can be further improved (Table 3). The urban slum context is complex social system. Communal toilet users must also work to build a more effective and sustainable management system for the maintenance of toilets and waste bins. The municipalities should invest in an improved waste collection and removal system for urban slums. In the interim, communities could find a sustainable way to pay a maintenance person to empty bins on a regular basis and post visible messages about the importance of toilet cleanliness. Toilet maintenance interventions will likely be more successful if they engage community leaders (landlords, teachers, or imams) to promote intervention behaviors, instill the importance of bin use and settle maintenance disputes (Table 3).

We view this study as the first in a series of actions that must be taken to help residents of low-income urban communities adapt to the direct effects of climate change on the urban environment, as well as increasing rural-to-urban migration accentuated by climate change and macro-economic forces. These phenomena will compound the vulnerability of low-income residents to heat stress, flooding, and breakdown of already fragile sanitary facilities [31].

## Abbreviations

BCC: Behavior change communication
DSK: Dustho Shastho Kendra
IBM-WASH: Integrated Behavioral Model- Water, Sanitation & Hygiene
NGO: Non-governmental organization
WASH: Water, Sanitation & Hygiene
WSUP: Water & Sanitation for the Urban Poor

## References

[1] Banks N, Roy M, Hulme D (2011). Neglecting the urban poor in Bangladesh: research, policy and action in the context of climate change. Environ Urban.

[2] Rahman MM, Atkins PJ, McFarlane C (2014). Factors affecting slum sanitation projects in Dhaka City: learning from the dynamics of social-technological-governance systems. Journal of Water Sanitation and Hygiene for Development.

[3] Heijnen M, Routray P, Torondel B, Clasen T (2015). Shared sanitation versus individual household latrines in urban slums: a cross-sectional study in Orissa, India. Am J Trop Med Hyg.

[4] Nelson KB, Karver J, Kullman C, Graham JP (2014). User perceptions of shared sanitation among rural households in Indonesia and Bangladesh. PLoS One.

[5] Heijnen M, Routray P, Torondel B, Clasen T (2015). Neighbour-shared versus communal latrines in urban slums: a cross-sectional study in Orissa, India exploring household demographics, accessibility, privacy, use and cleanliness. Trans R Soc Trop Med Hyg.

[6] Thye YP, Templeton MR, Ali M (2011). A critical review of technologies for pit latrine emptying in developing countries. Crit Rev Env Sci Tec.

[7] Jenkins M, Cumming O, Cairncross S (2015). Pit latrine emptying behavior and demand for sanitation Services in Dar Es Salaam, Tanzania. Int J Environ Res Public Health.

[8] Nakagiri A, Niwagaba CB, Nyenje PM, Kulabako RN, Tumuhairwe JB, Kansiime F (2016). Are pit latrines in urban areas of sub-Saharan Africa performing? A review of usage, filling, insects and odour nuisances. BMC Public Health.

[9] Alabaster G, Issaias I (2003). Removing human waste--the Vacutug solution. Habitat Debate.

[10] Opel A, Bashar MK (2013). Inefficient technology or misperceived demand: the failure of Vacutug-based pit-emptying services in Bangladesh. Waterlines.

[11] Brunner PH, Fellner J (2007). Setting priorities for waste management strategies in developing countries. Waste Manag Res.

[12] Dreibelbis R, Winch PJ, Leontsini E, Hulland KR, Ram PK, Unicomb L, Luby SP (2013). The integrated Behavioural model for water, sanitation, and hygiene: a systematic review of behavioural models and a framework for designing and evaluating behaviour change interventions in infrastructure-restricted settings. BMC Public Health.

[13] Nizame FA, Leontsini E, Luby SP, Nuruzzaman M, Parveen S, Winch PJ, Ram PK, Unicomb L (2016). Hygiene practices during food preparation in rural Bangladesh: opportunities to improve the impact of Handwashing interventions. The American Journal of Tropical Medicine and Hygiene.

[14] George CM, Monira S, Sack DA, Rashid M-u, Saif-Ur-Rahman KM, Mahmud T, Rahman Z, Mustafiz M, Bhuyian SI, Winch PJ (2016). randomized controlled trial of hospital-based hygiene and water treatment intervention (CHoBI7) to reduce cholera. Emerg Infect Dis.

[15] Rothstein JD, Leontsini E, Olortegui MP, Yori PP, Surkan PJ, Kosek M (2015). Determinants of Caregivers’ use and adoption of household water chlorination: a qualitative study with Peri-urban communities in the Peruvian Amazon. The American Journal of Tropical Medicine and Hygiene.

[16] Sridhar KS (2015). Is urban poverty more challenging than rural poverty?. A Review Environment and Urbanization Asia.

[17] Hossain S (2012). The informal practice of appropriation and social control - experience from a bosti in Dhaka. Environ Urban.

[18] Hanchett S, Akhter S, Khan MH. Water, sanitation and hygiene in Bangladeshi slums: an evaluation of the WaterAid-Bangladesh urban programme. Environment & Urbanization. 2003;15(2):43–56.

[19] Tumwebaze IK, Mosler H-J (2015). Effectiveness of group discussions and commitment in improving cleaning behaviour of shared sanitation users in Kampala, Uganda slums. Soc Sci Med.

[20] Alam MU, Winch PJ, Saxton RE, Nizame FA, Yeasmin F, Norman G, Masud AA, Begum F, Rahman M, Hossain K (2017). Behaviour change intervention to improve shared toilet maintenance and cleanliness in urban slums of Dhaka: a cluster-randomised controlled trial. Tropical Med Int Health.

[21] Saxton RE, Yeasmin F, Alam MU, Al-Masud A, Dutta NC, Yeasmin D, Luby SP, Unicomb L, Winch PJ: If I do not have enough water, then how could I bring additional water for toilet cleaning?! Addressing water scarcity to promote hygienic use of shared toilets in Dhaka, Bangladesh. Trop Med Int Health 2017. Jun 27. doi:10.1111/tmi.12914. 10.1111/tmi.12914 28656596

[22] Hackenbroch K, Hossain S (2012). “the organised encroachment of the powerful”-everyday practices of public space and water supply in Dhaka. Bangladesh Planning Theory & Practice.

[23] Hossain S (2011). Informal dynamics of a public utility: rationality of the scene behind a screen. Habitat International.

[24] Tumwebaze IK, Orach CG, Niwagaba C, Luthi C, Mosler HJ (2013). Sanitation facilities in Kampala slums, Uganda: users' satisfaction and determinant factors. Int J Environ Health Res.

[25] Wood W, Tam L, Witt MG (2005). Changing circumstances, disrupting habits. J Pers Soc Psychol.

[26] Burra S, Patel S, Kerr T (2003). Community-designed, built and managed toilet blocks in Indian cities. Environ Urban.

[27] Bandura A (1977). Self-efficacy: toward a unifying theory of behavioral change. Psychol Rev.

[28] Bandura A: Social learning through imitation. In: Nebraska Symposium on Motivation. edn. Edited by Jones MR. Oxford, UK: UNP; 1962: 211–274.

[29] Rashid SF, Michaud S (2000). Female adolescents and their sexuality: notions of honour, shame, purity and pollution during the floods. Disasters.

[30] Mahon T, Fernandes M (2010). Menstrual hygiene in South Asia: a neglected issue for WASH (water, sanitation and hygiene) programmes. Gend Dev.

[31] Alam M, Rabbani MDG (2007). Vulnerabilities and responses to climate change for Dhaka. Environ Urban.

